# Enhanced recovery protocols: compliance and outcome in elective vs. emergency colorectal surgery

**DOI:** 10.1007/s00423-026-04114-2

**Published:** 2026-06-24

**Authors:** Andres Heigl, Chiara Paganetti, Michael D. Honaker, Marionna Cathomas, Stephanie Taha-Mehlitz, Robert Rosenberg, Otto Kollmar, Anas Taha

**Affiliations:** 1 Department of Visceral Surgery, Cantonal Hospital Baselland, Liestal, Switzerland; 2 Department of Biomedical Engineering, Faculty of medicine, University of Basel, Basel, Switzerland; 3 Department of Surgery, Brody School of Medicine, East Carolina University, Greenville, NC USA

**Keywords:** Emergency surgery, Colorectal surgery, Enhanced recovery after surgery, ERAS, Patient outcomes

## Abstract

**Purpose:**

The feasibility and impact of Enhanced Recovery after Surgery (ERAS) protocols in emergency settings remain poorly understood due to patient instability, limited preoperative optimization, and inconsistent protocol adherence. Therefore, this study aimed to evaluate ERAS implementation in emergency colorectal surgery compared to elective cases.

**Methods:**

Using the ERAS^®^ Interactive Audit System (EIAS) database, a retrospective analysis was performed, encompassing all patients undergoing elective (*n* = 690) or urgent (*n* = 128) colorectal surgery between May 2016 and October 2024. Both groups were matched using 2:1 Propensity Scored Matching (PSM) and compared considering demographics, perioperative factors, and results. Postoperative complication rates, anastomotic leakage and compliance to the ERAS protocol were primary endpoints.

**Results:**

The number of elective cases was significantly higher than the number of emergency cases. Baseline characteristics and nature of the underlying disease appeared more favorable in the elective group. PSM analysis showed similar baseline characteristics between both groups. Within the emergency group the open approach was significantly higher compared to the elective group (*p* = 0.014). The compliance rate to ERAS was significantly lower in the emergency group compared to the elective group (71.12 ± 14.43 vs. 76.39 ± 12.59; *p* < 0.001). There was no significant difference in the rates of anastomotic leakage, major organ-specific complications, or mortality between both groups.

**Conclusion:**

ERAS implementation in emergency colorectal surgery is feasible despite lower compliance. Serious complications and mortality rates were similar to elective cases, highlighting the importance of developing specific ERAS protocols for emergency colorectal surgery.

**Supplementary Information:**

The online version contains supplementary material available at https://doi.org/10.1007/s00423-026-04114-2.

## Introduction

Enhanced Recovery after Surgery (ERAS) protocols in patients undergoing colorectal surgery are an evidence-based approach aimed at improving postoperative outcomes by reducing physiological stress during and after surgery through optimizing preoperative, intraoperative, and postoperative metrics [1]. The benefits of ERAS in colorectal surgery are well established. With good compliance, ERAS implementation results in reduced hospital length of stay (LOS), lower rate of complications, quicker recovery times, reduced opioid consumption, and higher patient satisfaction [2, 3]. Despite these benefits, the proper implementation of ERAS protocols has proved difficult, especially in rural and community hospitals. Insufficient equipment, poor multidisciplinary coordination, lack of experienced physicians, and poor patient compliance are some factors that hinder the effectiveness of ERAS [4–7].

These barriers to implementing ERAS properly are magnified when attempted for emergency colorectal surgeries due to multiple reasons. First, depending on the severity of presentation, it might not be possible to complete the full preoperative protocol components such as smoking cessation, nutritional optimization, anemia correction, and bowel preparation, all of these are factors enabling faster wound healing and lowering complication rates, leading to an overall shorter length of stay [8]. Furthermore, a considerable proportion of patients who emergently present for colorectal surgery are typically older individuals with a higher American Society of Anesthesiologists (ASA) score which translates to even higher physiological stress during surgery and longer recovery times [9–11]. In patients with bowel perforation complicated by peritonitis, implementing some postoperative ERAS components such as opioid-sparing analgesia, early mobilization, and enteral feeding might not be possible [12]. Hence, the compliance rate in patients who undergo emergency surgery is almost half the compliance rate of those who undergo elective surgery [13]. ERAS in an emergency setting will potentially still improve patient outcomes even with only partial compliance. For this reason, our institution implements ERAS in emergency surgeries whenever feasible. These factors highlight the importance of developing specific ERAS protocols to be developed and tested for emergency colorectal surgery.

Current literature on ERAS protocols in emergent cases remains limited [14, 15], as most studies investigate the efficacy of ERAS in elective surgery, where the setting is controlled and patients are thoroughly prepared in the preoperative phase. The scarcity of literature can be explained by the lack of trained staff in ERAS protocols and the severity of disease and heterogeneous comorbidities that are present in emergency settings. In contrast to elective surgery, there is limited time in emergencies to optimize patients preoperatively through nutritional, metabolic, or functional conditioning. Addressing these data gaps requires prospective multicenter studies that stratify emergency surgeries by severity, operation type, and patient characteristics to fully understand the outcomes of ERAS in high-risk settings as well as factors that alter these results. The results of these studies will guide consensus on evidence-based emergency-specific ERAS protocols that can be integrated into acute care to reduce complications, improve outcomes, and enhance patient satisfaction after emergency surgery.

Therefore, the present study evaluates the feasibility and outcome of ERAS implementation in emergency colorectal surgery compared to elective cases within a single institutional ERAS database. Factors associated with postoperative complications in the emergency setting will be analyzed.

## Methods

### Study design and patient selection

The implementation of the ERAS protocol for colorectal surgery patients was initiated at the Kantonsspital Baselland in May 2016. Since the start of the program, all patients undergoing elective or emergent colorectal surgery were included in the ERAS^®^ program because it was strongly hypothesized that participation in this protocol would significantly improve outcomes in both settings. All surgical procedures involving the colon and rectum were retrospectively retrieved and analyzed using the ERAS^®^ Interactive Audit System (EIAS) database.

All patients who underwent elective or emergent colorectal surgery for indications including neoplasia, diverticulitis, perforation, or inflammatory bowel disease between May 2016 and October 2024 were included. Emergency surgeries included urgent surgeries (within 24 h) or emergent surgeries (within 2 h). Further inclusion criteria were: open or laparoscopic approach, no robotic surgery; direct anastomosis in all cases, no protective stoma or discontinuous resection; no rectal surgery. Patients who did not give general consent or who could not be followed for more than three months after surgery were excluded, as the occurrence of postoperative complications can neither be confirmed nor excluded. Comprehensive preoperative data were collected, including age, sex, body mass index (BMI), Charlson Comorbidity Index, ASA score, nutritional status, smoking, surgical indication, type of procedure (right/left hemicolectomy, sigmoid resection, anterior resection of the rectum), protective ileostomy, and surgical approach, which compares the laparoscopic technique as minimally invasive surgery (MIS) and the open technique. Laboratory values such as hemoglobin levels and leucocyte count were recorded.

### Outcome measures

The primary endpoints of this study included postoperative complication rates and the occurrence of anastomotic leakage. Postoperative complications included medical (deep vein thrombosis, urinary tract infection, renal dysfunction, cerebrovascular lesions, cardiac dysfunction, etc.) and surgical complications (paralytic ileus, mechanical bowel obstruction, hematoma, wound dehiscence, wound infections, etc.). Secondary endpoints included operative time, LOS, compliance to ERAS, and the Comprehensive Complication Index (CCI). Compliance was calculated as percentage of the specific ERAS protocol elements successfully fulfilled per patient. The Clavien-Dindo classification was used for complications and divided into non-serious (grade I–II) and serious (grade III–V) complications. All data were collected and analyzed to compare outcomes between elective and emergent colorectal surgeries within the ERAS^®^ framework.

### Statistical analysis

Descriptive statistics were used to summarize baseline characteristics. Propensity score matching (PSM) analysis was performed to reduce baseline differences between patients undergoing elective and emergency surgery. Propensity scores were estimated using logistic regression analysis with emergency surgery status as the treatment variable. Matching was conducted using nearest-neighbor matching based on the logit of the propensity score, applying a 2:1 with baseline characteristics, such as age, sex, body mass index (BMI), ASA score, nutritional status, hemoglobin level, and leukocyte count (Figure S1), as these represented clinically relevant baseline characteristics potentially associated with postoperative outcomes and ERAS compliance. Covariate balance before and after matching was assessed using standardized mean differences (SMDs). Matching substantially improved the balance of most baseline variables, with post-matching SMDs below 0.1 for age, sex, BMI, ASA score, nutritional status, and hemoglobin level, indicating adequate balance between groups. Residual imbalance remained for leukocyte count (post-matching SMD = 0.478). Categorical variables were presented as counts and percentages, while continuous variables were presented as mean ± standard deviation. For continuous variables, between-group differences were evaluated using the Mann-Whitney U test for two groups or the Kruskal-Wallis test for more than two groups; for categorical variables, the chi-square or Fisher’s exact test were used as appropriate. P-values smaller than 0.05 were regarded as statistically significant. Figures were created with Python.

A multivariate logistic regression analysis was performed using ordinary least squares models on the entire cohort using overall postoperative complications as the dependent variable and clinically relevant baseline and operative variables as covariates. Missing data among predictor variables were addressed using k-nearest neighbor (KNN) imputation (k = 5) with distance-weighted neighbors. Imputation was restricted to covariates only, including categorical and continuous predictors.

## Results

### Baseline characteristics

The analysis included 816 patients, of which 690 were elective and 128 were emergency colorectal surgeries. Emergency surgery patients presented with significantly different preoperative risk profiles, including higher ASA scores (*p* = 0.036), lower hemoglobin levels (*p* < 0.002), elevated leukocyte counts (*p* < 0.001), and poorer nutritional status (*p* < 0.001). The baseline characteristics after PSM matching are shown in Table 1. Demographic factors such as sex, age, BMI, ASA, and nutritional status were comparable between groups. However, leukocyte count (*p* = 0.003) remained different between both groups.

**Table 1:** Baseline characteristics of patients (after PSM) undergoing elective versus emergency colorectal surgery

Variable	Sub-category	Elective surgery [*N* = 252]	Emergency surgery [*N* = 126]	*p*-value	SMD
Sex				0.913	0.024
	Female	*N* = 119(47.2%)	*N* = 58(46.0%)		
	Male	*N* = 133(52.8%)	*N* = 68(54.0%)		
Age (years)		67.18 ± 14.13	67.29 ± 16.06	0.621	0.008
Body mass index		27.02 ± 5.28	27.17 ± 5.02	0.831	0.029
Hemoglobin level (g/dL)		12.16 ± 2.20	12.31 ± 2.24	0.408	0.069
Leukocyte count (10^9/L)		9.50 ± 2.86	11.27 ± 4.88	**0.003**	0.478
ASA score				0.156	0.061
	ASA 1	*N* = 3(1.2%)			
	ASA 2	*N* = 69(27.4%)	*N* = 44(34.9%)		
	ASA 3	*N* = 175(69.4%)	*N* = 77(61.1%)		
	ASA 4	*N* = 5(2.0%)	*N* = 5(4.0%)		
Total points nutritional status				**0.002**	0.048
	No		*N* = 5(4.0%)		
	Yes, malnourished	*N* = 13(5.2%)	*N* = 2(1.6%)		
	Yes, normal status	*N* = 209(82.9%)	*N* = 98(77.8%)		
	Yes, risk of malnutrition	*N* = 30(11.9%)	*N* = 21(16.7%)		

### Operative characteristics

Operative characteristics differed significantly between groups (demonstrated in Table 2). Emergency procedures were most frequently performed via the MIS approach (51.6%), followed by the open approach (48.4%). This pattern is similar to the elective cases, but with a much lower proportion of open cases (34.9%) and a higher proportion of MIS cases (65.1%). Elective and emergency cases more often involved sigmoid resections (44.4% and 58.7%, respectively); this is followed by right hemicolectomy (35.7% vs. 29.4%, *p* = 0.005). An anastomosis was done in all the procedures included in the PSM matching. Despite being insignificant, a protective ileostomy was more commonly used in the emergency group (13.5%) as compared to the elective cases (8.3%). Operative time was slightly shorter in emergency cases (205.37 ± 59.43 min vs. 221.90 ± 66.77 min), though the difference did not reach statistical significance (*p* = 0.07).

**Table 2:** Intraoperative characteristics of patients (after PSM) undergoing elective versus emergency colorectal surgery

Variable	Sub-category	Elective surgery [*N* = 252]	Emergency surgery [*N* = 126]	*p*-value
Operation time (min)		221.90 ± 66.77	205.37 ± 59.43	0.070
Surgical approach group				**0.014**
	MIS	*N* = 164(65.1%)	*N* = 65(51.6%)	
	Open	*N* = 88(34.9%)	*N* = 61(48.4%)	
Main procedure name				**0.005**
	Anterior resection of rectum	*N* = 32(12.7%)	*N* = 4(3.2%)	
	Ileocaecal/right hemicolectomy	*N* = 90(35.7%)	*N* = 37(29.4%)	
	Left hemicolectomy	*N* = 18(7.1%)	*N* = 11(8.7%)	
	Sigmoid resection	*N* = 112(44.4%)	*N* = 74(58.7%)	
Protective ileostomy		*N* = 21(8.3%)	*N* = 17(13.5%)	0.183

## Postoperative characteristics

**Fig. 1:**
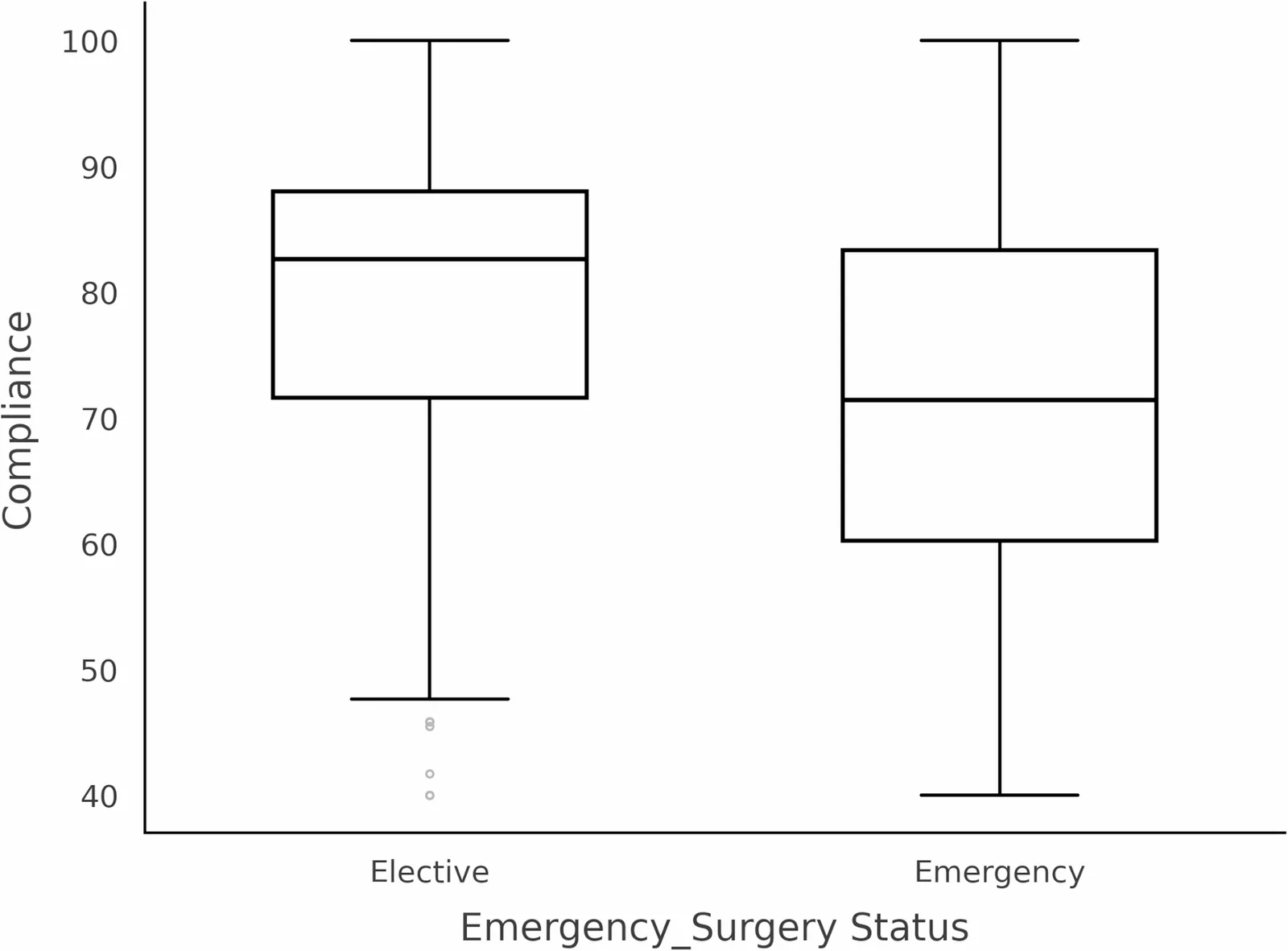
Perioperative compliance in elective and emergency surgery

ERAS compliance was significantly lower in emergency settings, with a rate of 71.12%±14.43 compared to the elective group (76.39%±12.59) as shown in Fig. 1. Postoperative outcomes demonstrated comparable data between the two groups. The overall complication rate, though not significant, was higher among emergency patients (38.1% vs. 34.5%, *p* = 0.50). Similarly, the CCI indicated a greater percentage of non-serious complications in the emergency group (31% vs. 23.4%), but this was not statistically significant (*p* = 0.21). In addition, the LOS was almost identical between the two groups (10.76 ± 8.37 vs. 10.38 ± 7.17, *p* = 0.26). The rates of anastomotic leakage and major organ-specific complications (pulmonary, cardiac, renal, gastrointestinal) did not differ significantly between the two groups (Table 3). Furthermore, complications were further subcategorized into wound infection, reoperation rate, bleeding, paralytic ileus, or respiratory, cardiac, anesthetic, or gastrointestinal complications. However, these results did not yield a significant difference in any category (supplementary materials, Table S1).

**Table 3:** Postoperative characteristics of patients (after PSM) undergoing elective versus emergency colorectal surgery

Variable	Sub-category	Elective surgery [*N* = 252]	Emergency surgery [*N* = 126]	*p*-value
Length of stay (days)		10.38 ± 7.17	10.76 ± 8.37	0.256
Total complications				0.497
	No	*N* = 165(65.5%)	*N* = 78(61.9%)	
	Yes	*N* = 87(34.5%)	*N* = 48(38.1%)	
Complication severity				0.205
	No	*N* = 166(65.9%)	*N* = 78(61.9%)	
	Non-serious complications	*N* = 59(23.4%)	*N* = 39(31.0%)	
	Serious complications	*N* = 27(10.7%)	*N* = 9(7.1%)	
Compliance (compliant)		76.39 ± 12.59	71.12 ± 14.43	**< 0.001**
Anastomotic leakage				0.645
	No	*N* = 240(95.2%)	*N* = 123(97.6%)	
	Yes, drained percutaneously	*N* = 1(0.4%)		
	Yes, radiological diagnosis with no intervention	*N* = 1(0.4%)		
	Yes, reoperated	*N* = 10(4.0%)	*N* = 3(2.4%)	
Discharged to				0.541
	Home	*N* = 178(70.6%)	*N* = 92(73.0%)	
	Hospital or Institution	*N* = 68(27.0%)	*N* = 33(26.2%)	
	No	*N* = 6(2.4%)	*N* = 1(0.8%)	

### Multivariate analysis

In the multivariate analysis, higher leukocyte count (β = 0.011, *p* = 0.037), lower hemoglobin level (β = −0.019, *p* = 0.035), and increasing age (β = 0.003, *p* = 0.038) were independently associated with postoperative complications. Surgical approach group was also independently associated with postoperative complications (β = 0.230, *p* < 0.001). Among the surgical procedures, anterior resection of the rectum was independently associated with postoperative complications (β = 0.212, *p* < 0.001), whereas ileocaecal/right hemicolectomy, left hemicolectomy, and sigmoid resection were not significantly associated with complication risk after adjustment. Emergency surgery status itself was not independently associated with postoperative complications after adjustment (β = 0.015, *p* = 0.752). Other investigated covariates, including sex, ASA score, nutritional status, and BMI, were not independently associated with complication risk (Table 4).

**Table 4:** Multivariate statistics using baseline variables as covariates and total complications as the target

Covariates	beta	*P*-value	CI lower	CI upper
Sex	−0.042	0.192	−0.106	0.021
ASA score	−0.033	0.343	−0.101	0.035
Total points nutritional status	−0.061	0.129	−0.139	0.018
Emergency Surgery	0.015	0.752	−0.077	0.106
Age (years)	0.003	**0.038**	0.000	0.005
Body mass index	0.004	0.208	−0.002	0.011
Hemoglobin level (g/dL)	−0.019	**0.035**	−0.037	−0.001
Leukocyte count (10^9/L)	0.011	**0.037**	0.001	0.021
Surgical approach group	0.230	**0.000**	0.155	0.304
Procedure: Anterior resection of rectum	0.212	**0.000**	0.098	0.325
Procedure: Ileocaecal/right hemicolectomy	0.042	0.407	−0.058	0.142
Procedure: Left hemicolectomy	0.030	0.632	−0.094	0.155
Procedure: Sigmoid resection	0.028	0.581	−0.071	0.127

## Discussion

This study compares the perioperative characteristics of elective and emergency colorectal surgeries under the ERAS framework. Although one-third of colorectal surgeries are non-elective and ERAS is strongly believed to benefit patients even in an emergency setting, current literature on ERAS in emergent cases remains largely limited [14, 15]. In this study, patients undergoing emergency surgeries had persistently higher ASA scores and leukocyte counts and poorer nutritional status after matching, which typically reflect the indications that prompt emergency surgeries, such as perforation, sepsis, or obstruction. Hence, these patients are not optimized prior to the procedure and usually have chronic comorbidities at presentation.

The emergency group showed a predominance of laparoscopic cases, highlighting the increased utility of MIS even under critical care settings. However, it also showed a significantly higher rate of open technique as compared to the elective group, which could be attributed to the challenges posed by the urgent nature of the emergency cases. These consist of increased inflammation leading to greater tissue friability and distorted anatomy, often necessitating open access and limiting the feasibility of minimally invasive techniques [16]. Nonetheless, the gradual advancement of MIS approaches in emergency surgeries has shown promise to achieve improved patient outcomes through increased adherence to the ERAS protocol. In the literature, there has been a clear trend of the benefits of MIS in emergency cases as compared to open techniques; older studies showed no significant difference in the outcomes between these groups [17, 18], while recent meta-analyses have proved shorter LOS, reduced complications, and overall improved patient outcomes in emergency MIS cases [19–21]. This is attributed to the improved learning curve of laparoscopic surgery and the increased proportion of experienced surgeons.

The compliance rate with the ERAS protocol was significantly lower in the emergency group compared to the elective cases. However, the rate remains remarkably high compared with previous studies that have assessed the compliance rate in non-elective settings. For instance, Roulin et al. [22] demonstrated in a prospective study of emergency colon resections that ERAS compliance was 77% in elective cases versus 57% in emergency settings [22]. After applying the ERAS protocol to emergency surgeries, Bisagni et al. [15] revealed a 50% postoperative compliance rate. They reported notable improvements despite lower compliance: Overall morbidity and mortality stayed the same, LOS decreased, and the use of laparoscopic procedures increased from 12% to 40% [15]. In the emergency group, patients could only benefit from the intraoperative and the postoperative components of the protocol, as preoperative optimization is mostly not feasible. This supports the concept of a modified ERAS approach for emergency colorectal surgery, focusing on the intraoperative and postoperative components rather than abandoning the protocol entirely.

In the present study, patients of the emergency group had a similar complication rate and comorbidities compared to the elective group after the baseline characteristics were matched. Hence, multivariate analysis including the whole dataset showed no independent factor associated with complications other than the leukocyte count, which likely reflects the pathophysiological stress of the presenting disease. After subcategorizing each type of complication, there were still similar outcomes between both settings. This finding contradicts most studies in the literature, which have consistently shown higher complication rates in emergency cases. Therefore, it suggests the potential advantage of ERAS in emergency settings, as the compliance rate in this study was higher than in previous studies [23, 24]. Anterior resection of the rectum was independently associated with higher complication rates. This is likely secondary to the increased technical complexity and the restricted pelvic space in rectal procedures compared with colonic surgeries [25]. Studies have shown that rectal surgeries have higher rates of anastomotic leakage and pelvic sepsis; hence, increased morbidity and lower survival rates [26, 27].

There are various limitations of this study. First, its retrospective design introduces selection and information biases that are difficult to completely control. Second, the differences in outcomes may be attributed to the fact that patients undergoing emergency colorectal surgery typically have poorer baseline characteristics, including greater comorbidities and higher inflammatory markers. Third, the preoperative components of the ERAS protocol are often not fully applicable in the emergency setting, as preoperative optimization and patient education are frequently challenging to achieve. As a result, compliance differed throughout the perioperative period, and it is still unclear which particular components (preoperative, intraoperative, or postoperative) were the most impactful.

## Conclusion

This study shows that the complication rate of patients undergoing elective and emergency colorectal surgery is not different within a clinical setting of ERAS for all patients. This benefit is attributed to the high compliance rate to the implementation of ERAS, which was much higher than in the literature. These findings support the notion that enhanced recovery principles as ERAS protocols should be introduced to emergency colorectal surgery to enhance recovery and maintain surgical quality. Future research should be done to implement specific emergency enhanced recovery protocols and stratify each component to measure the most impactful measure for improved outcomes. As similar complication rate can be attributed to ERAS in the present study, however, this finding can only be confirmed by further prospective enhanced recovery protocol studies.

## Supplementary Information

Below is the link to the electronic supplementary material.

Supplementary File 1 (DOCX 4.15 MB)

## Data Availability

All data supporting the findings of this study are available within the paper and its supplementary material.
