# Supplementary material for: Enhanced recovery protocols: compliance and outcome in elective vs. emergency colorectal surgery
Source: Langenbecks Arch Surg. 2026 Jun 24;411(1):226. doi: 10.1007/s00423-026-04114-2 (PMC13550093; doi:10.1007/s00423-026-04114-2)
Supplement: Supplementary file 1 [file 423_2026_4114_MOESM1_ESM.docx]

**Supplementary Materials**

Figure S1: Study flow chart - Co-variables for propensity score matching were Sex, Age, BMI, Hemoglobin level, Leukocyte count, ASA score and Total points nutritional status.

Table S1: Postoperative Complications (after 2:1 matching)

| **Variable** | **sub-category** | **Elective Surgery [N=252]** | **Emergency Surgery [N=126]** | **p-value** |
| --- | --- | --- | --- | --- |
| Anastomotic leakage |  |  |  | 0.645 |
|  | No | N=240(95.2%) | N=123(97.6%) |  |
|  | Yes, drained percutaneously | N=1(0.4%) |  |  |
|  | Yes, radiological diagnosis with no intervention | N=1(0.4%) |  |  |
|  | Yes, reoperated | N=10(4.0%) | N=3(2.4%) |  |
| Complication after surgery (Pulmonary) |  |  |  | 1.000 |
|  | No | N=235(93.3%) | N=117(92.9%) |  |
|  | Yes | N=17(6.7%) | N=9(7.1%) |  |
| Complication after surgery (Cardial) |  |  |  | 0.449 |
|  | No | N=248(98.4%) | N=122(96.8%) |  |
|  | Yes | N=4(1.6%) | N=4(3.2%) |  |
| Complication after surgery (Gastrointestinal) |  |  |  | 0.745 |
|  | No | N=221(87.7%) | N=109(86.5%) |  |
|  | Yes | N=31(12.3%) | N=17(13.5%) |  |
| Complication after surgery (Kidneys) |  |  |  | 0.730 |
|  | No | N=248(98.4%) | N=125(99.2%) |  |
|  | Yes, oliguria | N=1(0.4%) |  |  |
|  | Yes, renal failure | N=3(1.2%) | N=1(0.8%) |  |
| Complication after surgery (Urogenital) |  |  |  | 0.554 |
|  | No | N=250(99.2%) | N=126(100.0%) |  |
|  | Yes | N=2(0.8%) |  |  |
| Complication after surgery (Central nervous system) |  |  |  | 1.000 |
|  | No | N=251(99.6%) | N=126(100.0%) |  |
|  | Yes | N=1(0.4%) |  |  |
| Complication after surgery (Wound Complications) |  |  |  | 1.000 |
|  | No | N=247(98.0%) | N=123(97.6%) |  |
|  | Yes | N=5(2.0%) | N=3(2.4%) |  |
| Complication after surgery (Thromboembolic) |  |  |  | 1.000 |
|  | No | N=251(99.6%) | N=126(100.0%) |  |
|  | Yes | N=1(0.4%) |  |  |
| Complication after surgery ((central) line associated bloodstream) |  |  |  | 1.000 |
|  | No | N=251(99.6%) | N=126(100.0%) |  |
|  | Yes | N=1(0.4%) |  |  |
| Complication after surgery (Anaesthetic) |  |  |  | 1.000 |
|  | No | N=251(99.6%) | N=126(100.0%) |  |
|  | Yes | N=1(0.4%) |  |  |
| Complication after surgery (Gastrointestinal haemorrhage) |  |  |  | 1.000 |
|  | No | N=251(99.6%) | N=125(99.2%) |  |
|  | Yes | N=1(0.4%) | N=1(0.8%) |  |
| Complication after surgery (Infectious complications) |  |  |  | 0.853 |
|  | No | N=227(90.1%) | N=115(91.3%) |  |
|  | Yes | N=25(9.9%) | N=11(8.7%) |  |
| Complication after surgery (Mechanical bowel obstruction) |  |  |  | 0.669 |
|  | No | N=248(98.4%) | N=125(99.2%) |  |
|  | Yes | N=4(1.6%) | N=1(0.8%) |  |
| Complication after surgery (Nausea or vomiting) |  |  |  | 0.847 |
|  | No | N=230(91.3%) | N=116(92.1%) |  |
|  | Yes | N=22(8.7%) | N=10(7.9%) |  |
| Complication after surgery (Obstipation or diarrhoea) |  |  |  | 0.669 |
|  | No | N=248(98.4%) | N=125(99.2%) |  |
|  | Yes | N=4(1.6%) | N=1(0.8%) |  |
| Complication after surgery (Pain) |  |  |  | 0.191 |
|  | No | N=247(98.0%) | N=120(95.2%) |  |
|  | Yes | N=5(2.0%) | N=6(4.8%) |  |
| Complication after surgery (Postoperative excessive haemorrhage) |  |  |  | 1.000 |
|  | No | N=247(98.0%) | N=124(98.4%) |  |
|  | Yes | N=5(2.0%) | N=2(1.6%) |  |
| Complication after surgery (Postoperative paralytic ileus) |  |  |  | 0.833 |
|  | No | N=234(92.9%) | N=118(93.7%) |  |
|  | Yes | N=18(7.1%) | N=8(6.3%) |  |
| Complication after surgery (Prolonged postoperative sedation) |  |  |  | N/A |
|  | No | N=252(100.0%) | N=126(100.0%) |  |
| Complication after surgery (Surgical complications) |  |  |  | 0.138 |
|  | No | N=206(81.7%) | N=111(88.1%) |  |
|  | Yes | N=46(18.3%) | N=15(11.9%) |  |
